# ADDRESSING LANGUAGE BARRIERS: PERSPECTIVES INTO THE ROLE OF FAMILY CAREGIVERS FOR OLDER ASIAN AMERICANS

**DOI:** 10.1093/geroni/igae098.0637

**Published:** 2024-12-31

**Authors:** Minakshi Raj, Laura Quintero

**Affiliations:** University of Illinois Urbana-Champaign, Champaign, Illinois, United States; University of Illinois Urbana-Champaign, Champaign, Illinois, United States

## Abstract

Language barriers influence the healthcare experience. Limited communication due to language differences impacts rapport establishment and decision-making processes, often increasing caregivers’ care burden by making them responsible for language interpretation when such service is not offered in healthcare settings (HCS). This study aimed to describe the challenges that caregivers face regarding navigating language barriers in HCS with or on behalf of their older Asian American relatives. Ninety-six caregivers completed a cross-sectional online survey of questions on a) language-related characteristics (e.g., at-home language, other languages spoken), b) language-related experiences in HCS, c) demographic information (e.g., age, residence, immigration status), and d) caregiving characteristics (proximity to care recipient, hours of caregiving per week, and types of support provided). Most respondents (72%) reported that a language other than English was the primary language spoken at home, while 41% of care recipients understand English “not well” (25%) or “not at all” (16%). In HCS, 54% of care recipients wanted interpretation services, with 30% “always” having it. Among types of interpretation, caregivers and relatives performed this task more frequently (53%). Regarding challenges, caregivers referred care recipients’ aging and cognitive decline, medical and technical vocabulary not translatable to native languages, and lack of training are the issues they experience in navigating language barriers in HCS. Despite significant cultural diversity, the United States continues to experience gaps in providing equitable health services to linguistically diverse individuals. It is necessary to develop support strategies to improve healthcare experiences and reduce the additional burden of language barriers on caregivers.

